# Net survival for colorectal cancer in Cuiabá and Várzea Grande (state of Mato Grosso), Brazil

**DOI:** 10.3332/ecancer.2021.1196

**Published:** 2021-03-02

**Authors:** Christiane Maria Meurer Alves, Pedro Cainelli de Oliveira Prado, Ronaldo Rocha Bastos

**Affiliations:** 1 Programa de Pós Graduação em Saúde Coletiva, Universidade Federal de Juiz de Fora, Rua José Lourenço Kelmer, s/n – São Pedro, Juiz de Fora, 36036-900, Brazil; 2 Departamento de Estatística, Universidade Federal de Juiz de Fora, Rua José Lourenço Kelmer, s/n – São Pedro, Juiz de Fora, 36036-900, Brazil

**Keywords:** colorectal cancer, survival analysis, Brazil

## Abstract

In studies of cancer survival, Population-Based Cancer Registries (PBCRs) can provide an overview of the disease for places that have this source of information available. In Brazil, PBCR is officially available in 22 state capitals and 8 cities in the interior of the country. PBCR data from Cuiabá and Várzea Grande, state of Mato Grosso, in Midwestern Brazil, were used to estimate the survival rate of colon (C18), rectosigmoid junction (C19) and rectum (C20) cancer cases diagnosed in 2000–2009 according to the International Classification of Diseases, 10th Revision. Five-year survival rate was estimated by the unbiased and consistent net survival estimator, which is used in the country estimates of the global surveillance of cancer survival programme CONCORD Group, for all cases, and also by sex, age group, diagnosis period and place of residence. The probability of death and the number of years of life lost to illness were also estimated. The estimated standardised 5-year survival rate for colorectal cancer was 45.46% (95% CI: 43.09%–47.96%) in the cities of Cuiabá and Várzea Grande. There was no difference between the curves when the survival rate was assessed by diagnostic period (2000–2004 and 2005–2009), sex, age group or city of residence. The gross 5-year probability of death from the disease was 51.2%, accounting for 6.4% of the gross probability of death from other causes, with 2.07 being the years of life lost to illness. The results obtained for Cuiabá and Várzea Grande are compatible with survival rates estimated for Brazil in the CONCORD study, but demonstrate the need to identify reasons why we continue to have low survival rates when compared to most countries involved in the global study mentioned. The results may reflect late diagnosis, difficult access and delays in starting treatment.

## Introduction

Recent data show that cancer incidence and mortality are increasing worldwide [1]. These measures, along with survival, are common indicators in epidemiology and, when reliable, can provide an overview of the impact of this disease on the geographic region of interest. They can also be used to evaluate disease control strategies [2].

The efficient and effective planning of cancer control programmes depends on the creation of indicators. In Brazil, where neoplasms were the second leading cause of death in 2018, second only to diseases of the circulatory system [3], these indicators are created by using information from Cancer Registries and the Mortality Information System (MIS) [3, 4].

The country has two types of Cancer Registries – the Hospital-Based Registry, which has a mainly clinical role, i.e., a resource to monitor patient care and the quality of the work provided by the hospital, and the Population-Based Registry, which is intended to find out more about the population in its coverage area [4]. The Population-Based Registry is found in 22 state capitals and 8 cities in the interior of the country [4].

Colorectal cancer is a known important disease which may be related to hereditary syndromes or family history [5, 6]. However, in most cases, it occurs sporadically and is associated with inappropriate lifestyle and behaviour [7, 8]. Increased mortality and incidence of this disease have been observed in Brazil [9–13].

Cancer survival data in several countries around the world, including Brazil, have been presented by the CONCORD Programme [14]. Six Population-Based Cancer Registries (PBCRs) in the country submit their data for analysis, among which is the Registry of Cuiabá that includes data from Cuiabá and Várzea Grande. Cuiabá is the capital of the state of Mato Grosso in Midwestern Brazil [15]. For 2020, the state has an estimated adjusted colorectal cancer incidence rate of 10.19 cases/100,000 for men and 12.64 cases/100,000 for women [4].

Registries such as those that provide data for the CONCORD Programme often obtain information on the cause of death only through death certificates [14]. To deal with potential errors/distortions in the information thus obtained, the concept of relative survival was developed, where deaths from other causes would not be considered a censoring event. Therefore, knowing the cause of death would not be required. Relative survival would be a ratio for the survival seen in the cohort of cancer patients and the expected survival for a comparable group, but without the disease [16].

The methods that have been generally used to calculate the relative survival rate are Ederer I, Ederer II and Hakulinen. These three methods differ in terms of the time during which the matched individuals are considered at risk to calculate the expected survival rate [16].

More recently, another method has been proposed and is being used in the CONCORD Programme – the Pohar Perme method or net survival. In this method, a hypothetical situation is estimated in which the study disease would be the only possible cause of death. The relevant risk is broken down into risk from the disease and risk from other causes [17].

Risk breakdown is possible when the times to death from the disease and from any other cause are independent conditions. It is assumed that the risk of death from other causes is given by the population’s risk of mortality and that the presence of the disease makes the risk of death higher than that observed in the population. The survival rate derived from the excess risk is the net survival – an important estimator in comparing populations, as it is independent of the population risk [17].

When comparing the Ederer II method to the Pohar Perme (net survival) method, both were found to perform well, except in long follow-up studies with a >5-year censoring [18].

The purpose of this study was to evaluate the 5-year net survival of colorectal cancer cases in the cities of Cuiabá and Várzea Grande from 2000 to 2009 using data from the PBCR in order to more specifically understand the status of the disease in the covered area. The municipalities of Cuiabá and Várzea Grande concentrate just over 20% of the population of the state of Mato Grosso [3]. Besides, the Cuiabá and Várzea Grande PBCR meets the quality criteria established by the National Cancer Institute: more than 70% of the cases with pathological anatomical diagnosis, less than 20% of the cases notified only by the death verification service, ignored age in less than 10% of the cases and location not specified in less than 10% of cases [4].

## Materials and methods

This study was conducted with the database provided by the Cuiabá and Várzea Grande PBCR and reviewed all cases that had been registered as colon cancer (C18.0-C18.9), rectosigmoid junction cancer (C19) and rectal cancer (C20) according to the International Classification of Diseases, 10^th^ Revision [19], from 2000 to 2009, in order to estimate the 5-year net survival.

Using a unique code in the R software [20], the database was standardised, i.e., all capital letters, removal of accent marks, among others, enabling the deterministic search for duplicate cases (name, mother’s name, date of birth and, if present, date of death). Subsequently, the Reclink [21] software was used for a probabilistic search for duplicity (name, mother’s name, date of birth and address). The same programmes were used to compare the registry data with those of the identified MIS obtained from the State Health Department of the state of Mato Grosso for 2000–2014. At this stage, potential updates on the death registers were sought so as to estimate the 5-year net survival for all cases included.

The 5-year net survival was calculated for all cases and also by sex, age group, diagnosis period (2000–2004 and 2005–2009), city of residence and also for the combined information of sex and age group, city and age group. The Pohar Perme [17] net survival estimator was chosen – a consistent non-parametric estimator for relative survival, the same used by the CONCORD Group [14]. As a preliminary exploratory analysis, the total number of cases was used to compare the relative survival methodologies known as Ederer I and Ederer II.

Five age groups were considered: 0–44 years old, 45–54 years old, 55–64 years old, 65–74 years old and ≥75 years old, to enable the subsequent standardisation of the survival rate according to the International Cancer Survival Standard criteria, where colorectal cancer is part of Group 1 for tumours that increase with age. Corazziari *et al* [22] also determine that the weight assigned to the 15–44-year-old age group can be safely assigned to the 0–44-year-old age group, since colorectal cancer is among those for which the portion of childhood cancer would be insignificant [22].

The life tables for population standardisation were taken from the CONCORD Group, which uses the linear interpolation method [23, 24]. However, availability of data in Cuiabá was only for women for the entire period. Thus, the life table of Goiânia, another capital city of Midwestern Brazil, whose registration data were used in the CONCORD Group, was selected for men. The use of a life table from another nearby location with similar characteristics is not expected to have any major impact on the result [25, 26].

The relsurv [27] package of the R software [20] was used to compare the relative survival and net survival estimation techniques, to apply the chosen methodology and also to estimate the gross probability of death from the study disease and death from other causes, also estimating the number of years of life lost to illness. The package also allowed the comparison of the survival curves by the log-rank-type test proposed by Grafféo et al [27] and applied elsewhere by Perme and Pavillič [28]. As for other log-rank tests, this test was only performed when the risks were proportional, as this is where the most reliable results are seen [28]. A significance level of 0.05 was used to compare the curves.

The project was approved by the Human Research Ethics Committee of the Federal University of Juiz de Fora (CAAE: 66979017.0.0000.5147; Res. # 2.046.497).

## Results

**Figure 1.**
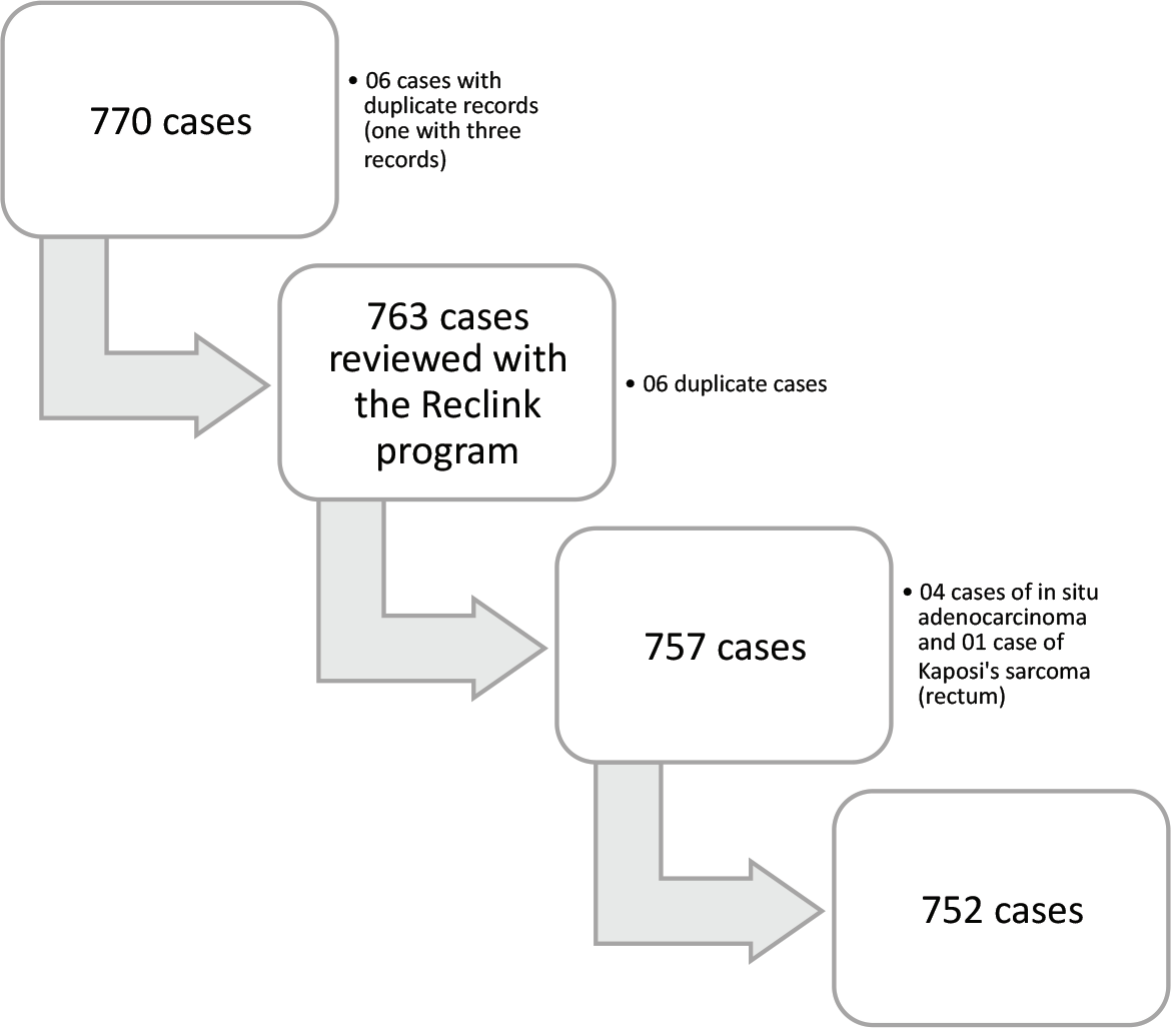
Flowchart of the deterministic (R software) and probabilistic (Reclink III) search in the database.

The original file contained 770 records, but duplicates were found after the deterministic and probabilistic search, with 757 cases remaining. When reviewing these cases, four of them were coded as D01.0, i.e., *in situ* adenocarcinoma, which were generally excluded from the survival analyses, and one case was diagnosed as C46.7 – Kaposi’s sarcoma – and did not meet the inclusion criteria as previously defined (C18–C20). These five cases were also excluded, with 752 cases then remaining in the base. Cases identified only through the death check service, corresponding to 60 cases in the relevant base, were also excluded, as the survival time of these patients is not known, and its use might affect the estimates. Therefore, 692 cases were used for the survival study (Figure 1).

The description of the main features in the reviewed database for the survival rate study can be seen in absolute numbers and percentages in Table 1.

The comparison of methods using all cases showed that the estimated 5-year net survival was 47.7% (95% CI: 43.4%–51.8%), which is very close to that observed for the Ederer I (48.5%; 95% CI: 44.2%–52.6%) and Ederer II (47.6%; 95% CI: 43.4%–57.7%) methods. After standardisation by age group, the resulting net survival was 45.5% (95% CI: 43.1%–47.9%).

When assessing net survival by diagnostic period – 2000–2004 being the first period, and 2005–2009 the second period – despite the numerical difference between point estimates, i.e., 44.4% in the first period (95% CI: 38.3%–50.4%) and 50.6% in the second period (95% CI: 44.7%–56.2%), the difference between the curves was not statistically significant (*p* = 0.16), even when stratified by age group (*p* = 0.2).

**Figure 2.**
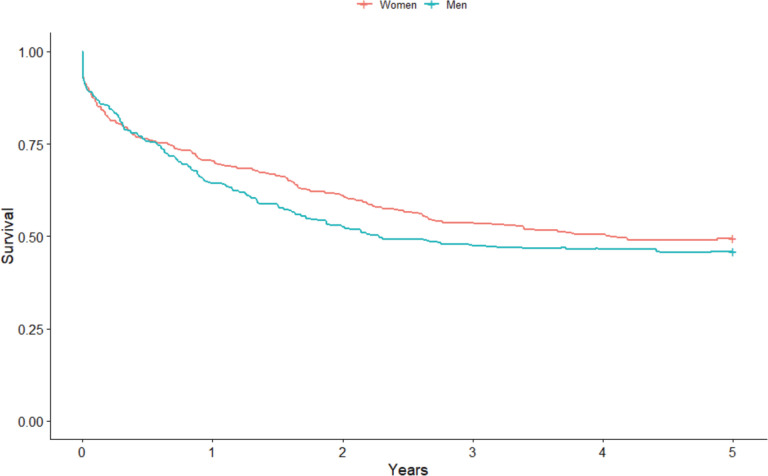
Non-standardised 5-year sex-based colorectal cancer net survival in the cities of Cuiabá and Várzea Grande, state of Mato Grosso, Brazil – cases from 2000 to 2009.

Considering sex, women had a higher survival point estimate of 49.5% (95% CI: 46.3%–55.1%) compared to men (45.9% (95% CI: 39.7%–51, 8%)) (Figure 2). Again, the difference between the curves was not statistically significant (*p* = 0.3), even when stratified by age group (*p* = 0.22). When standardised, the estimated net survival for women was 47.0% (95% CI: 43.9%–50.3%) and 44.0% for men (95% CI: 40.8%–47.6%). As for the city of residence, the survival rate was greater in the point estimate for those in the city of Cuiabá (49.3%; 95% CI: 44.5%–54.0%) compared to Várzea Grande (42.6%; 95% CI: 33.9%–50.9%), but there was no statistically significant difference between the curves (*p* = 0.13), even after stratification by age group (*p* = 0.11). The standardised survival rate was 45.9% (95% CI: 43.3%–48.7%) for the city of Cuiabá and 45.0% (95% CI: 39.9%–50.9%) for Várzea Grande.

**Figure 3.**
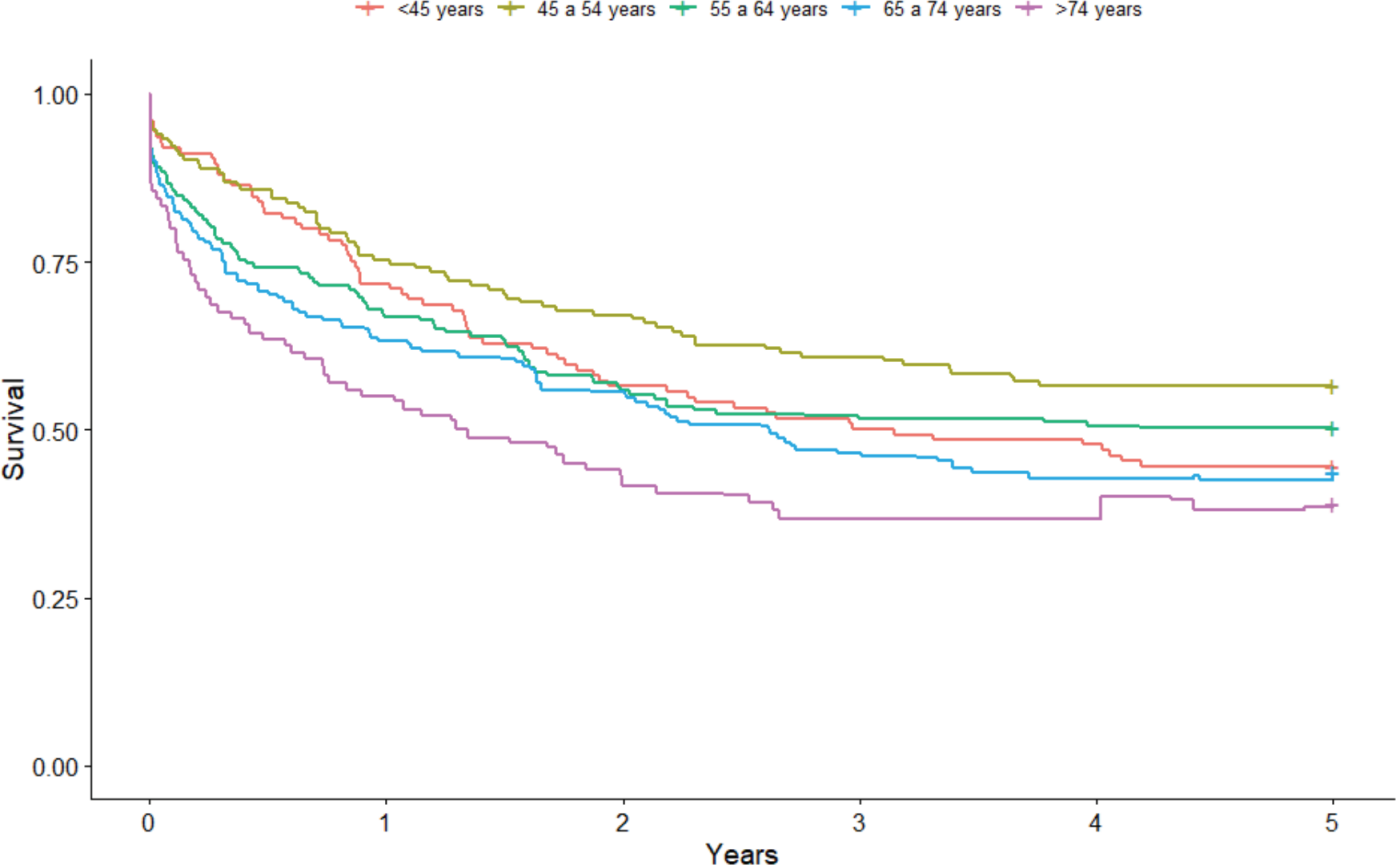
Non-standardised 5-year age group-based colorectal cancer net survival in the cities of Cuiabá and Várzea Grande, state of Mato Grosso, Brazil – cases from 2000 to 2009.

As for the age group, the highest point survival rates were seen in the 45–54-year-old (56.6%; 95% CI: 47.8%–64.4%) and 55–64-year-old (55.6%; 95% CI: 41.8%–58.3%) age groups. The lowest survival rates were seen in the oldest age groups of 65–74 years old (43.6%; 95% CI: 34.7%–52.2%) and >74 years old (39.0%; 95% CI: 24.7%–53.0%). Those aged <45 years old showed an intermediate result with a 5-year survival rate of 44.7% (95% CI: 35.7%–53.2%) (Figure 3).

When stratified by sex and age group, the highest point estimates for the 5-year survival rate were found in the 45–54-year-old age group, both for women – 58.8% (95% CI: 48.1%–71.9%) – and men – 54.6% (95% CI: 44.2%–67.5%).

**Figure 4.**
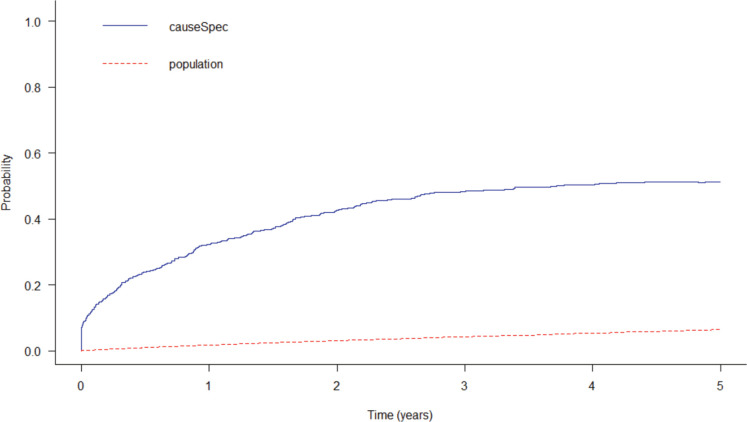
Gross probability of death from colorectal cancer and population death in Cuiabá and Várzea Grande, state of Mato Grosso, Brazil over a 5-year period.

After 5 years since diagnosis, the probability of death from the study disease was 51.2% and 6.4% of the gross probability of death from other causes, with 2.07 being the years of life lost to illness. It is possible to see that the gross probability of death from the disease increases initially and becomes more stable over time (Figure 4).

## Discussion

The standardised estimated colorectal cancer net survival rate in the study population was 45.5%, which is lower than that seen in most countries involved in the CONCORD Programme where the survival for this pathology exceeds 50% in the 2010–2014 period [14]. This result reflects the need of improvement in the Brazilian health system to achieve similar survival rates. On the other hand, these findings are compatible with what was observed for Brazil in the aforementioned study, with survival estimates of 44.5%, 50.6% and 48.3% for colon cancer, and 37.7%, 45.7% and 42.4% for rectal cancer for the three periods of 2000–2004, 2005–2009 and 2010–2014, respectively [14].

As seen in the CONCORD Group study for Brazil, a potential improvement in the survival rate, even if not statistically significant, is suggested for the study cities between the first and second study periods – 44.4% and 50.6%, respectively.

Sex appears to be an independent prognostic predictor in colorectal cancer, with women having a longer survival rate; this could be attributed to genetic, hormonal, immune or environmental factors [29]. The study consistently indicated a higher net survival rate for women in the point estimate, although this difference was not statistically significant.

In addition to adopting healthy habits – which is up to the individual – in order to reduce the risk of a number of pathologies, including colorectal cancer [7, 8], health care systems should be able to offer and enable primary prevention by screening for precursor lesion resection (polyps), enable early diagnosis, if possible, and also effective treatment. The relatively low survival rate observed shows that there is a need for improvement in health education and in the screening and treatment system.

As for screening, it is known that, if well indicated, it can reduce mortality and increase survival [30]. There are several types of screening, including colonoscopy, rectosigmoidoscopy and faecal occult blood test. Screening is recommended to start at the age of 50 in Brazil [4], but the American Cancer Society considers it as a ‘qualified recommendation’ from the age of 45 [31].

Brazil does not have an organised cancer screening programme. The implementation of population screening was not considered feasible and cost-effective; however, the importance of sending warning signs to the population and health care providers was recognised, as well as providing immediate access to diagnostic means when the disease is suspected [32]. A recent study showed that population screening based on quantitative faecal immunochemical test may be an opportunity in Brazil for early diagnosis [33], but a major barrier would still be how to deal with increased demand for definitive diagnosis [34].

In the state of Mato Grosso, only 10%–20% of the population receives Supplementary Health care [35]. The rest of the population will only be able to receive the diagnosis and all stages of treatment from the Unified Health System (*Sistema Único de Saúde*) – universal health care created in the 1988 Constitution. The Unified Health System has as principles the universality of access, equal care and equity. Actions should be organised in an integrated, regionalised and hierarchical manner [36]. Its creation represented an increase in the supply of services, but regional differences, underfunding, changes in the age structure of the population and changes in its health conditions compromise its effectiveness [37]. Silva *et al* [38] showed that the provision of specialised services for cancer is still deficient in the country. The low survival rate in the study region may reflect the difficulty in accessing health services, when existent, due to social, economical, cultural and information barriers.

In an overview of some risk and protection factors related to colorectal cancer [7], an increase can be seen in overweight and obesity for both sexes in the population of Cuiabá from 2006 to 2018. On the other hand, there was a decrease in physical inactivity in the city. For the consumption of alcoholic beverages, there was a slight increase in the total population from 18.9% (95% CI: 17.1%–20.6%) in 2006 to 19.8% (95% CI: 17.4%–22.1%) in 2018. However, the result was due to increased consumption by women, since for men there was a decrease in the intake of alcoholic beverages [39, 40].

As for the state of Mato Grosso, there is a high consumption of red meat and a high proportion of adults classified as insufficiently active [41]. These are risk factors unrelated to survival, but it is important to recognise the profile of the population involved in this study.

A 14% decrease in the overall survival rate is estimated for every 4 weeks of delay in the adjuvant treatment [42]. Other studies involving colon cancer found similar results only [43, 44]. Specifically for patients with stage III colon cancer, impaired survival was observed in delays longer than 8 weeks after surgery. This delay in starting the adjuvant treatment was associated to age >65 years, among other factors [43]. Difficulties in the course between surgical treatment and adjuvant treatment, when required, may be associated with the results found in this study. In addition, the lowest net survival rate results were estimated for patients aged 65–74 (43.6%) and >74 (39%). This result may be partly related to treatment delays, as well as the administration of less intense treatments than indicated for individuals over 70 years of age [45], even when there are benefits from the treatment [46].

In addition to considering the patient’s course to identify potential delays that could compromise the survival rate, the type of treatment also deserves attention. This study showed an improvement, albeit not statistically significant, for the estimated number of cases in the second study period (2005–2009). There have been many developments in the field of colorectal cancer treatment in recent years, including the incorporation of new drugs [47, 48]. However, it is not possible to say whether there was any influence and how extensive it was in a population-based study [43].

Regarding the methodology, the use of data from this study to compare three methods (Ederer I, Ederer II and net survival) showed similar results when the observation is made for a short period (5 years), but the Pohar Perme method would be considered an unbiased estimator when censoring is non-informative and the calculation uses continuous time [18].

Even though there are limitations to the use of PBCR data, such as a lack of detailed information on the therapeutic course, surgical procedure, staging and complementary treatment, these are results considered to be key measurements to assess the health care system effectiveness in the management of cancer patients, as it can give an overview that does not depend on age, social condition, comorbidities and stage of the disease at diagnosis [49]. Relative survival methodologies, and in particular the net survival estimation methodology, were developed precisely for this type of data [14, 16, 17].

Some data, which were incomplete in the studied PBCR, such as education, race, occupation and extent of the disease, if complete, could enrich the analysis and indicate groups that might need more attention.

## Conclusions

Data such as time between symptoms and diagnosis, time between diagnosis and treatment, laterality, histology, extension, type of treatment, possible complications, associated pathologies could contribute to a better understanding of the results, but should be the subject of other studies. Complementary studies, like hospital-based or cohort-based studies, on the itinerary course of colorectal cancer patients in Cuiabá and Várzea Grande can also help to identify flaws in the system and would contribute to establishing a better cause and effect relationship if carried out for the region’s population.

In any case, the existence of a PBCR shows an interest in serving the population, since the data produced by the registry can be a source of information for epidemiological studies in an attempt to identify populations at risk and measure the effectiveness of cancer prevention and control programmes [4].

The development of awareness and warning campaigns about the disease and risk factors can somehow impact the survival rate by determining earlier diagnoses and a consequent increase in survival.

## List of abbreviations

ICD-10, International Classification of Diseases, 10^th^ Revision;

MIS, Mortality Information System;

PBCRs, Population-Based Cancer Registries.

## Conflicts of interest

The authors declare no conflicts of interest.

## Funding

None.

## Ethics approval

This exploratory study was approved by the Human Research Ethics Committee of the Federal University of Juiz de Fora (CAAE: 66979017.0.0000.5147; Res. # 2.046.497).

## Authors’ contributions

All authors participated in all stages of the study and approved the article before submission.

## Figures and Tables

**Table 1.** Features of the Cuiabá and Várzea Grande (MT) Cancer Registry database, ­Brazil, for colorectal cancer, 2000–2009.

Features	Absolute numbers (%)
**Sex**	
Male	347 (50.14)
Female	344 (49.71)
N.A.	1 (0.14)
**Age**	12–100 years old (mean 58.5 years old; median 59 years old)
**Color**	
Yellow	2 (0.29)
White	264 (38.15)
Indigenous	2 (0.29)
Brown	217 (31.36)
Black	40 (5.78)
N.A.	167 (24.13)
**City**	
Cuiabá	530 (76.59)
Várzea Grande	162 (23.41)
**Topography**	
(ICD 10) Colon (C18)	391 (56.50)
Sigmoid (C19)	81 (11.70)
Rectum (C20)	220 (31.80)
**Extension**	
Local	277 (40.03)
Metastasis	132 (19.07)
N.A.	283 (40.90)
**Age group**	
<45 years old	123 (17.77)
Aged 45–54	150 (21.68)
Aged 55–64	163 (23.55)
Aged 65–74	167 (24.13)
Aged ≥74	83 (11.99)
N.A.	6 (0.87)

N.A., Not available; ICD, International Classification of Diseases
